# Active Craniospinal Tensioning (ACT): Axial Spinal Traction for Glymphatic Modulation

**DOI:** 10.7759/cureus.101796

**Published:** 2026-01-18

**Authors:** Huan-Wei Chen

**Affiliations:** 1 Chiropractic, Private Practice, Vancouver, CAN

**Keywords:** active craniospinal tensioning (act), amyotrophic lateral sclerosis, axial spinal traction, biomechanical neuromodulation, cerebrospinal fluid dynamics, diffusion tensor imaging analysis along the perivascular space (dti-alps), dural tensioning, glymphatic system, neurodegenerative disease, pelvis-stabilized axial spinal traction (psast)

## Abstract

The glymphatic system is a clearance pathway that facilitates convective exchange between cerebrospinal fluid (CSF) and interstitial fluid, and is increasingly implicated in the pathophysiology of neurodegenerative and neuroinflammatory disorders. However, translation of glymphatic physiology into intentional, noninvasive biomechanical interventions remains limited. Building on a previously introduced biomechanical framework that proposed axial spinal traction as a method to modulate CSF and glymphatic circulation, practitioner-applied pelvis-stabilized axial spinal traction (PSAST) has been characterized as a controlled prototype, although its reliance on professional administration and structured setup may constrain deployment in population-level or preventive contexts.

This report introduces active craniospinal tensioning (ACT) as a translational, participant-driven extension of the established axial traction-glymphatic modulation framework, specifically designed for ambulatory populations. ACT employs a voluntary squat maneuver combined with a standardized overhead anchor system to generate controlled axial tension along the craniospinal axis, while preserving the same dural tensioning and craniospinal coupling principles described in prior axial traction models. By shifting force generation from practitioner-applied loading to participant-regulated loading, ACT presents a scalable approach for exploratory preventive application under supervised conditions.

ACT is theoretically framed for individuals exhibiting reduced perivascular water diffusivity, such as those with lower diffusion tensor imaging analysis along the perivascular space (DTI-ALPS) indices. This report provides a practical implementation framework for ACT and supports future hypothesis-driven investigation of biomechanical modulation of CSF and glymphatic circulation using advanced neuroimaging biomarkers.

## Introduction

The glymphatic system is a clearance pathway that facilitates convective exchange between cerebrospinal fluid (CSF) and interstitial fluid, thereby contributing to the removal of metabolic waste, inflammatory mediators, and proteinaceous byproducts from the central nervous system. Together with the rediscovery of dural lymphatic vessels, recognition of the glymphatic pathway has substantially reshaped contemporary understanding of brain fluid dynamics and parenchymal waste clearance. Impairment of glymphatic function has been increasingly implicated in the pathophysiology of neurodegenerative diseases and a broader spectrum of disorders associated with disrupted fluid homeostasis, including Alzheimer’s disease, Parkinson’s disease, traumatic brain injury, amyotrophic lateral sclerosis (ALS), Huntington’s disease, major depressive disorder and other psychiatric conditions, multiple sclerosis, neuroinflammatory disorders, and substance use disorders [1].

Despite these advances, the translation of glymphatic physiology into intentional, noninvasive, mechanically grounded interventions capable of modulating CSF-glymphatic circulation remains an unmet clinical challenge. In particular, while pharmacologic and lifestyle correlates of glymphatic function have been explored, few approaches have addressed whether biomechanical manipulation of the craniospinal system itself could be deliberately leveraged to influence glymphatic transport.

To address this gap, we previously introduced the concept that controlled axial spinal traction can modulate CSF dynamics and glymphatic circulation through craniospinal hydraulic coupling. This hypothesis was first motivated by a preliminary longitudinal clinical observation in which pelvis-stabilized axial spinal traction (PSAST) was applied to a patient with probable bulbar-onset ALS. In that case, bulbar and respiratory function were sustained for more than 21 months, an outcome inconsistent with expected disease progression and sufficient to justify further mechanistic investigation [2].

Building on this observation, we subsequently formalized the biomechanical and anatomical rationale for axial spinal traction-based glymphatic modulation in a dedicated technical report [3]. That work explicitly proposed axial spinal traction as a novel, practitioner-applied biomechanical strategy for modulating CSF and glymphatic circulation, grounded in the anatomical continuity and mechanical responsiveness of the craniospinal dural sac [4]. To the best of the author’s knowledge, this represented the first explicit formulation of axial spinal traction as a method for intentional modulation of CSF and glymphatic dynamics, and it established conceptual priority for this traction-based framework. That report further extended the hypothesis to multiple categories of neurodegenerative disease characterized by impaired glymphatic function, positioning axial spinal traction as a unifying biomechanical paradigm rather than a disease-specific intervention.

Within this framework, PSAST was presented as a controlled prototype intervention designed to explore the effects of transient craniospinal elongation and dural tensioning on CSF dynamics and glymphatic circulation. However, its reliance on externally applied force, professional administration, and specialized setup may limit scalability, accessibility, and deployment in preventive or population-level contexts.

To address these practical constraints without altering the underlying biomechanical mechanism or conceptual foundation, a simplified and participant-driven implementation is required. In this technical report, active craniospinal tensioning (ACT) is introduced as a translational extension of the previously defined axial spinal traction framework, rather than as a novel or independent intervention. ACT preserves the core principle established in prior work, that controlled axial elongation and dural tensioning can influence CSF-glymphatic dynamics, while reconfiguring force generation into an active, self-regulated maneuver.

ACT shifts axial spinal traction from a passive, practitioner-dependent application to an active biomechanical process in which controlled axial loading is generated through a voluntary partial squat. This approach enables reproducible craniospinal traction forces using low-cost, non-motorized components, without relying on external traction devices. As such, ACT may be particularly suitable for ambulatory populations and for exploratory preventive applications. This preventive relevance is underscored by accumulating evidence that glymphatic dysfunction may precede overt clinical manifestations of neurodegenerative disease by one to two decades [5].

To facilitate objective evaluation of axial spinal traction-based interventions, diffusion tensor imaging analysis along the perivascular space (DTI-ALPS) is proposed as a surrogate biomarker of glymphatic function [6,7]. Reduced DTI-ALPS indices have been reported across a wide range of neurodegenerative, psychiatric, and autoimmune disorders [6-19], positioning DTI-ALPS as a pragmatic tool for assessing whether axial traction interventions, including both PSAST and ACT, produce measurable alterations in glymphatic-related fluid dynamics. When applied at early disease stages or in presymptomatic populations, post-intervention elevations in DTI-ALPS indices could provide objective support for biomechanical modulation of glymphatic function and justify further investigation into potential effects on disease trajectories.

The purpose of this report is to describe the practical implementation and biomechanical rationale of ACT and to extend the previously established axial spinal traction-glymphatic modulation framework to broader, ambulatory populations. ACT is not presented as a stand-alone therapy or a substitute for medical management, but as a hypothesis-generating, adjunctive, and potentially preventive approach. By clearly delineating the conceptual lineage, mechanistic continuity, and safety considerations of ACT, this report aims to support reproducibility, encourage independent verification, and facilitate future investigations into biomechanical modulation of CSF and glymphatic circulation in brain health and disease.

## Technical report

Active craniospinal tensioning (ACT)

Procedural Configuration and Equipment

ACT is a participant-driven maneuver designed to generate brief, controlled axial traction along the craniospinal axis. The following procedural description outlines an experimental implementation designed for reproducibility in various ambulatory settings.

In this configuration, traction is achieved using a fixed overhead anchor point secured to a structurally sound doorframe (wood or metal). The anchor consists of a steel screw-and-washer assembly, providing a stable load-bearing point capable of supporting forces exceeding those generated during body-weight-derived traction. A wide webbing strap, approximately 5 cm in width and comparable in tensile strength to an automotive seatbelt, is fashioned into a continuous loop to serve as the traction interface. The strap is positioned over the anchor assembly to create stable overhead support while distributing the load across the occipital region to minimize localized pressure.

All equipment is inspected before use. While ACT is self-applied, the protocol requires the presence of a safety spotter to provide safety assistance and ensure participant stability. Alternative anchoring systems or harness designs may be utilized, provided they maintain equivalent load-bearing capacity and stability. The setup described herein is selected for its low cost, global availability, and feasibility in clinical or observational research environments.

Biomechanical Implementation

To initiate traction, the participant faces the doorframe and positions the webbing strap to cradle the occiput. A thin folded towel may be interposed between the strap and the head to provide cushioning and prevent focal compressive forces on the occiput and ears. The strap must be oriented to extend upward and forward from the occiput, covering or passing over the pinnae (external ears), allowing the participant to firmly clasp the two ends together at the forehead. The participant uses both hands to seal the webbing loop; this manual grip serves a critical structural function by locking the strap against the occipital bone. Crucially, the hands must not exert downward pulling force; their role is strictly to maintain the closed loop, creating a fail-safe mechanism.

Axial spinal traction is realized entirely through a voluntary partial squat. As the participant lowers their body weight, the strap transmits the load to the occiput, elongating the craniospinal axis. It is a mandatory safety requirement that the strap must not cross the anterior neck nor be placed beneath the chin to avoid airway or carotid compression. The traction force, estimated at 50% to 80% of body weight, is sustained for a duration of two to five seconds per session. A minimum threshold of two seconds is required to allow for the gradual, voluntary application of axial force and to ensure the longitudinal tension fully propagates through the length of the spinal dural sac. The maneuver is designed to be performed consistently, repeated one to three times daily. This efficiency results in a total daily time commitment of less than 60 seconds.

Preliminary Safety and Feasibility Data

Longitudinal feasibility testing is ongoing, spanning eight years in a single-subject model (the author) to evaluate long-term tolerability. Throughout this duration of daily adherence, no significant or lasting adverse events have been recorded. Minor, transient neck soreness has been occasionally noted, resolving spontaneously without intervention. These data are reported for descriptive feasibility purposes and do not imply therapeutic efficacy.

Physiological limits are defined by the interaction between the equipment interface and cranial anatomy. Due to the webbing strap’s width (approximately 5 cm), the effective contact area on the occiput is relatively small, resulting in inevitable partial compression of the suboccipital soft tissues and underlying vascular structures during loading. It has been observed that when traction exceeds approximately five seconds, this focal compression may precipitate transient presyncopal symptoms, such as lightheadedness, tinnitus, or visual dimming.

**Figure 1:**
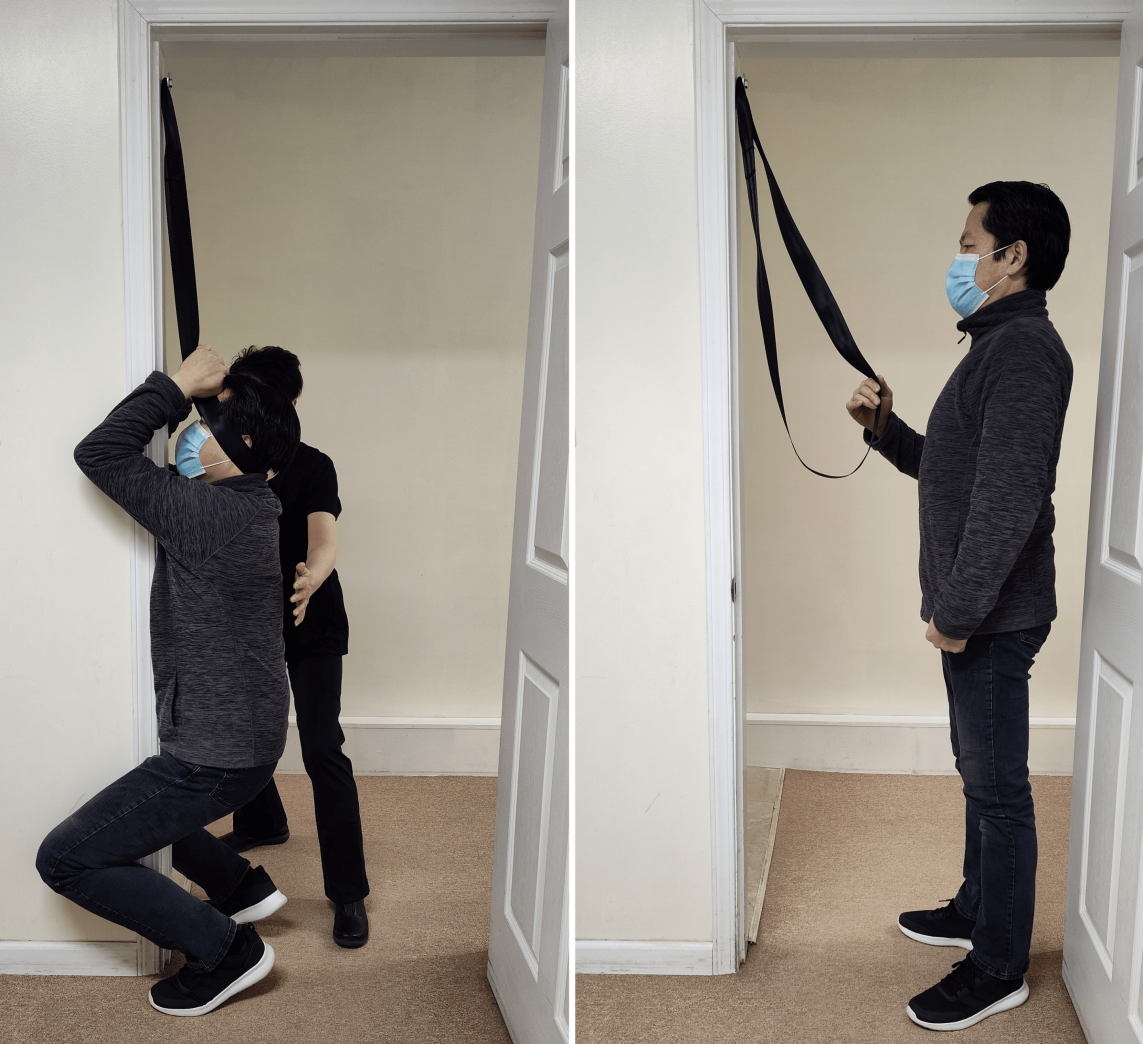
Active craniospinal tensioning (ACT) The strap is oriented anteriorly and superiorly from the occiput, passing over the ears to be clasped at the forehead. A thin folded towel may be interposed between the strap and the head to provide cushioning and prevent focal compressive forces on the occiput and ears. Hands are used only to close the loop, not to pull. Axial traction is generated by a voluntary partial squat, transmitting 50% to 80% of body weight to the occiput. The protocol requires a single two- to five-second hold per session, repeated one to three times daily; this efficiency results in a total daily time commitment of less than 60 seconds. A safety spotter acts as a mandatory precaution against instability Image credit: Author (as participant) and consenting model (as safety spotter)

Therefore, the five-second maximum duration is established not merely as a suggestion, but as a mandatory safety control to prevent hemodynamic or neural compromise. By restricting each session to this short window, the protocol avoids the onset of these symptoms while still delivering the intended axial tension. If any neurological, vascular, or presyncopal symptoms occur even within this timeframe, the maneuver must be terminated immediately. A prompt medical evaluation should be sought if such symptoms persist (Figure 1).

Biomechanical rationale

The theoretical basis for ACT relies on the unique vector of its loading force. While conventional spinal motions, such as flexion, extension, and rotation, involve angular displacement around segmental axes, axial elongation represents a longitudinal cranio-caudal tensile motion along the vertical axis of the spine. This specific loading direction is mechanically distinct because the spine is predominantly subjected to gravitational and muscular compression during daily activities. Unlike standard angular motions, meaningful axial decompression cannot be achieved through independent muscle activity without external assistance. Within this framework, ACT employs a fixed overhead anchor and a webbing strap to provide the necessary mechanical support for longitudinal distraction.

The biomechanical basis for axial spinal traction as a potential modulator of CSF and glymphatic circulation has been detailed in prior technical literature [3]. In summary, the craniospinal compartment, enclosed by the intracranial dura mater and its caudal continuation as the spinal dural sac, functions as a continuous hydraulic system permitting CSF movement across the foramen magnum. The spinal dura mater is firmly anchored near the foramen magnum superiorly and to the sacrum inferiorly, while the intervening segment retains limited longitudinal distensibility [4]. Within this framework, ACT is hypothesized to transiently elongate the vertebral column and tension the spinal dural sac, thereby generating momentary CSF pressure gradients within the spinal compartment.

During the active traction phase and subsequent release, these transient pressure gradients may propagate across the foramen magnum, producing a single-cycle dural pull-recoil stroke of CSF motion that could influence intracranial CSF exchange and glymphatic transport. Although this conceptual model was originally described in relation to practitioner-applied PSAST [3], the same craniospinal hydraulic principles are proposed to apply to ACT, insofar as the participant-driven maneuver produces brief, controlled cranio-caudal loading along the craniospinal axis. This proposed mechanism remains theoretical and requires experimental validation.

The following force estimates are derived from theoretical biomechanical principles rather than direct experimental measurement. It is postulated that an axial tensile load is transmitted to the occipital region via the holding strap. Because the hands clasp the strap ends to form a loop, the upper extremities are supported directly by the strap mechanism, effectively offloading their mass from the spinal column. Consequently, the maximal tensile force acting at the craniospinal junction is calculated as total body weight minus the mass of the head and the supported upper extremities. This resulting load is estimated at approximately 80% to 90% of total body weight. As the load is transmitted caudally, it is characterized by progressive attenuation; the maximal tensile force at the lumbosacral junction corresponds primarily to the body mass distal to the waist, representing approximately 50% to 60% of total body weight.

The magnitude of force generated during ACT is inherently constrained by body weight and voluntary squat control. Thus, the effective axial tensile load is estimated to fall within a range similar to PSAST, which is approximately 50% to 80% of total body weight. In the configuration described, traction is applied for short durations comparable to those used in practitioner-led PSAST protocols, typically lasting two to five seconds [3]. By utilizing brief, high-magnitude loading, ACT aims to induce potent mechanical perturbations of CSF while remaining within the limits of physiological tolerance.

From a fluid dynamics perspective, it is proposed that brief active loading within this estimated force range may be sufficient to induce transient changes in dural tension and CSF displacement. Such perturbations could secondarily influence glymphatic transport, which is driven by pressure gradients that facilitate advective flow along perivascular spaces [1]. While the physiological effects of ACT have not been directly measured, the similarity in loading duration and estimated force range supports the biomechanical plausibility of ACT as a lower-complexity, active analogue of PSAST [3] for ambulatory populations. This rationale provides a foundation for future experimental evaluation of ACT and PSAST using objective physiological and imaging-based surrogate measures of glymphatic function.

## Discussion

Safety considerations of ACT

The general safety considerations associated with axial spinal traction, including vascular, neurological, and structural contraindications, as well as comprehensive screening principles, have been detailed in the prior technical report introducing PSAST [3]. To focus on the translational evolution of this method, this report refers readers to the previously published framework for established clinical and professional application standards. While the prior framework provides the necessary detail on structural risks and contraindications, the present report expands upon the specific, active safety mechanisms intrinsic to the self-regulated ACT maneuver, such as the participant's sensory-motor feedback loop and the manual fail-safe mechanism.

While ACT shares the underlying biomechanical principles of PSAST, it differs fundamentally in its mode of delivery, introducing distinct safety advantages through its active nature. First, because traction forces are participant-driven and realized specifically through a voluntary squat, the potential for unintended or excessive force application is inherently limited by the user's physical capacity; the maximum tensile load is physically restricted to the available gravitational load. Second, the participant’s continuous sensory-motor feedback loop serves as a primary safety mechanism. The dual effort required to manually clasp the webbing loop closed and maintain the controlled squat naturally regulates the intensity of the tensile load, allowing the participant to terminate the maneuver instantly upon sensing discomfort. Third, it is proposed that this manual interface creates a functional fail-safe mechanism. The configuration requires the participant to use both hands to clasp the strap ends together near the forehead, effectively closing the loop that cradles the occiput. Because the structural integrity of the traction setup depends entirely on this active manual closure, any loss of consciousness or cessation of voluntary motor effort would cause the hands to separate. This action immediately opens the webbing loop and automatically releases the strap from the occiput, preventing prolonged suspension in the event of syncope.

Despite these intrinsic safety features, the shift toward an active protocol introduces specific supervision requirements. ACT necessitates rigorous professional screening and individualized instruction to ensure the participant understands the precise physical mechanics of the maneuver. Furthermore, the presence of a safety spotter is mandatory to safeguard against accidental falls, ensure the precise occipital placement of the strap, and provide immediate physical assistance if the participant experiences transient neurological or vascular symptoms. This requirement for a safety spotter is a critical risk-mitigation strategy to manage the potential for injury from a loss of balance.

Finally, the reliance on self-regulation dictates specific inclusion criteria. Participants must maintain the cognitive and physical capacity to terminate the squat immediately upon any sign of discomfort or presyncope. This requirement distinguishes ACT as a protocol optimized for ambulatory individuals and preventive applications; conversely, non-ambulatory patients or those with cognitive impairment remain candidates for the supervised, practitioner-led passive PSAST described previously [3].

Glymphatic modulation and DTI-ALPS as a surrogate biomarker

The present report does not claim therapeutic efficacy for ACT in any specific disorder. Rather, its potential relevance to a range of glymphatic-related conditions is based on published evidence demonstrating reduced DTI-ALPS indices, which are increasingly regarded as a surrogate imaging biomarker of glymphatic function [6,7].

Neurodegenerative diseases, psychiatric illnesses, substance-use disorders, autoimmune or neuroinflammatory diseases, and neurodevelopmental disorders have consistently been reported to exhibit reduced DTI-ALPS indices, suggesting impaired glymphatic circulation [6-19]. Because these changes often manifest in the presymptomatic or early stages of disease, they identify a critical window for intervention. It is hypothesized that ACT and PSAST may mechanically modulate CSF and glymphatic dynamics, potentially elevating DTI-ALPS indices toward normative levels. Such improvements could be detectable immediately following a single treatment session or manifest as a cumulative effect over a longitudinal therapeutic course (e.g., three to six months). Consequently, these patient populations represent rational candidates for hypothesis-driven investigation. Future studies incorporating pre- and post-intervention DTI-ALPS imaging, alongside physiological and clinical measures, are required to determine whether ACT and PSAST produce measurable, sustained increases in glymphatic surrogate biomarkers and to clarify their role within broader, multimodal prevention strategies (Table 1).

**Table 1:** Representative conditions associated with reduced DTI-ALPS index DTI-ALPS is a diffusion-based magnetic resonance imaging metric proposed as a non-invasive surrogate biomarker of glymphatic system function. Reduced DTI-ALPS indices are interpreted as reflecting decreased perivascular water diffusivity and potentially impaired glymphatic transport. The conditions listed are representative examples reported in the literature and are not intended to be exhaustive DTI-ALPS: diffusion tensor imaging analysis along the perivascular space; CSF: cerebrospinal fluid

Category	Representative disorders (DTI-ALPS ↓)	Proposed mechanism	References
Neurodegenerative disorders	Alzheimer’s disease; Parkinson’s disease; amyotrophic lateral sclerosis; Huntington's disease	Reduced perivascular water diffusivity, suggesting impaired glymphatic clearance across multiple neurodegenerative conditions	[6-10]
Psychiatric and stress-related disorders	Major depressive disorder; post-traumatic stress disorder	Glymphatic dysfunction associated with mood disturbance, chronic stress, and sleep fragmentation	[11,12]
Substance use disorders	Alcohol use disorder; opioid use disorder; cannabis use disorder	Substance-related impairment of perivascular diffusivity and CSF dynamics	[13-15]
Autoimmune/inflammatory disorders	Multiple sclerosis; systemic lupus erythematosus	Immune-mediated perivascular inflammation affecting CSF-interstitial fluid exchange	[16,17]
Neurodevelopmental disorders	Autism spectrum disorder; attention deficit hyperactivity disorder	Altered CSF dynamics and perivascular diffusivity during critical neurodevelopmental windows	[18,19]

Clinical utility and preventive application

A primary distinction of ACT is its potential utility in the presymptomatic phase of neurodegenerative and neuroinflammatory disorders. Current research suggests that impairment of the glymphatic system [1] leads to the accumulation of proteinaceous waste, such as amyloid-beta, a process that may precede the clinical symptoms of Alzheimer’s disease by 20 years [5].

While PSAST, as originally described by Chen (2025) [3], remains the preferred model for patients with advanced disease or limited mobility who require practitioner-applied force, ACT represents a translational, participant-driven extension of the PSAST framework, providing a scalable and active alternative for ambulatory individuals. The minimal cost and high accessibility of the ACT setup facilitate long-term adherence, which is critical for interventions aimed at maintaining brain fluid homeostasis over many years. By implementing ACT as a preventive neurological maintenance tool, ambulatory populations could support glymphatic clearance pathways during the critical window before irreversible parenchymal damage occurs, while preserving the conceptual lineage and biomechanical principles originally established by PSAST.

Contraindications, precautions, and disclaimer

Comprehensive contraindications, precautions, and general safety considerations associated with axial spinal traction have been described previously in the technical report introducing practitioner-applied PSAST [3], and these remain applicable to the present ACT framework. These considerations include, but are not limited to, appropriate professional screening, identification of vascular, neurological, or structural contraindications, and the avoidance of traction in individuals for whom axial loading may pose undue risk. To avoid redundancy, these previously described contraindications and precautions are not repeated here but should be considered integral to the application of ACT.

The methods described in this report represent exploratory biomechanical procedures intended solely for hypothesis generation and controlled application under appropriate professional screening and supervision. ACT is defined as a participant-driven maneuver designed for brief, voluntary tensioning and is not intended for suspension, prolonged loading, or any activity that may pose a risk of self-injury. Deviation from the described protocol, inappropriate application, or unsupervised use may result in serious injury.

The reliance on an active motor task, specifically the squat, requires that the individual possess the physical and cognitive capacity to immediately terminate the maneuver. Furthermore, the mandatory presence of a safety spotter serves as a critical safeguard to maintain stability and ensure proper equipment positioning. The author assumes no responsibility for adverse outcomes arising from use outside the conditions, parameters, and professional oversight described in this report.

## Conclusions

ACT represents a translational advancement in the application of biomechanical force for the modulation of central nervous system fluid dynamics. By transitioning from practitioner-led passive traction to a participant-driven, active maneuver, this protocol offers a scalable and accessible framework for exploring the mechanical influences of axial spinal traction on glymphatic circulation. It is proposed that the brief 'dural pull-recoil stroke' generated by this maneuver serves as an acute mechanical perturbation, theoretically capable of promoting transient CSF displacement and modulating the glymphatic system. The inherent simplicity of the described setup facilitates its potential use in broad ambulatory populations, particularly as a preventive strategy during presymptomatic phases. While the relationship between active axial elongation and enhanced waste clearance remains theoretical, the biomechanical plausibility of this mechanism provides a foundation for future investigation. Further studies utilizing advanced neuroimaging and surrogate biomarkers, such as the DTI-ALPS index, are required to clarify the physiological impact of axial spinal traction and its potential role in maintaining brain fluid homeostasis across a wide spectrum of disorders.
